# Interleukin-1β-Induced Inflammatory Signaling and Myelin-Related Alterations in Oligodendroglia and Schwann Cells in Multiple Sclerosis: Modulatory Effects of Ibuprofen and Flurbiprofen

**DOI:** 10.3390/cimb48090953

**Published:** 2026-09-18

**Authors:** Jagadeesh Narasimhappagari, Ling Liu, W. Sue T. Griffin

**Affiliations:** Department of Geriatrics, Donald W Reynolds Institute on Aging, University of Arkansas for Medical Sciences, Little Rock, AR 72205, USA; njagadeesh@uams.edu (J.N.); liuling@uams.edu (L.L.)

**Keywords:** neuroinflammation, Interleukin-1β, multiple sclerosis, Ibuprofen, Flurbiprofen

## Abstract

Neuroinflammation, read as elevated levels of Interleukin-1β (IL-1β) and of its downstream signaling cascade, has been shown to play a neuropathogenic role in numerous neurodegenerative diseases, including Alzheimer’s and Parkinson’s disease. Western blotting was used to determine the effects of IL-1β and its signaling cascade on Human Oligodendroglioma (HOG) and Schwann cells and to evaluate the potential therapeutic effects of Ibuprofen and Flurbiprofen on MS pathogenesis in vitro. IL-1β increased neuroinflammatory markers like TNFα, MyD88, βAPP, COX-2, and IL-6, as well as transcription factors like NFκB and STAT in HOG and Schwann cells, which in turn regulate the expression of other cytokines, resulting in the activation of the self-perpetuating Cytokine Cycle. The IL-1β treatment of HOG and Schwann cells decreased the levels of myelin Basic Protein (MBP) and synaptophysin while at the same time increasing βAPP. Autophagy markers like LAMP-2 and LC3B were downregulated. In contrast, treatment with Ibuprofen and Flurbiprofen effectively countered the harmful effects of IL-1β in MS. These in vitro studies show that IL-1β can play a possible detrimental role in facilitating neuropathogenesis, as seen in Alzheimer’s and Parkinson’s disease, and anti-inflammatory drugs Ibuprofen and Flurbiprofen have a potential role in modulating the neuroinflammation in MS.

## 1. Introduction

Multiple sclerosis (MS) is an inflammatory disease of the brain and spinal cord that manifests clinically with different temporal and pathologic patterns and results in numerous neurologic signs and symptoms. Immune cell invasion of the central nervous system in MS triggers key pathological events, including demyelination and axon loss, and requires communication among invading leukocytes, astrocytes, microglia, and neurons. Interleukin-1α and IL-1β are important prototypical proinflammatory cytokines that exert pleiotropic effects on numerous types of cells and play an important role in acute and chronic inflammatory pathways and a variety of autoimmune disorders. There are two IL-1β receptors, namely, IL-1 type 1 receptor (IL-1RI) and IL-1 type 2 receptor (IL-1RII). IL-1α and IL-1β signaling with the aid of IL-1RI binding to IL-1RII does not lead to cell signaling and is therefore considered a decoy receptor. When IL-1β binds to IL-1RI, a second receptor, named the IL-1 receptor accessory protein (IL-1RAcP), is recruited to the cell membrane to form a high-affinity receptor complex, resulting in the activation of intracellular signaling. A third IL-1 family member, the IL-1 receptor antagonist (IL-1RA or Anakinra), binds to IL-1 receptors and blocks IL-1β from interacting with its receptors, acting as a natural IL-1β inhibitor [1,2].

IL-1β plays an important homeostatic function by which it governs important mechanisms like feeding, sleep, and temperature [1]. However, the exacerbated production of IL-1β is involved in the pathophysiological changes that happen during numerous disease states, like rheumatoid arthritis, neuropathic pain, inflammatory bowel disease, osteoarthritis, vascular disease, MS, Alzheimer’s disease, Parkinson’s disease and Downs Syndrome [1,2,3]. IL-1β can also be released from different physiological cells like keratinocytes, fibroblasts, endothelial, neuronal and immune cells (mainly macrophages and mast cells), and glial cells in the periphery such as Schwann cells, but principally from microglia and astrocytes [1,2,3,4,5,6,7].

Myelin, which is an important membrane structure that surrounds the axon of nerve cells, acts as an insulating sheath that prevents the mutual interference between nerve impulses conducted by various nerve fibers while simultaneously acting to increase the impulse conduction velocity of nerve fibers, especially fast conduction via the Nodes of Ranvier [8]. Conversely, demyelinating diseases like MS destroy the myelin sheath, leading to the slowing of axonal signaling and the potential for axon rupture. The subsequent clearance of myelin debris by important cells like phagocytes, such as microglia in the brain and macrophages in the periphery, markedly affects the function of neurons and their axons by decreasing the speed of axonal transmission in diseased tissues, and in this way promoting the development and continuation of neurodegenerative disease [9]. The myelin sheath of the axons in the brain is mainly derived from oligodendrocyte precursor cells (OPCs) that are broadly distributed in the gray and white matter mostly in the adult brain, and execute an important role in the process of the regeneration and repair of myelin in chronic demyelinating diseases like MS [10,11]. However, the inflammatory reaction and oxidative stress prevailing in these pathological disorders often negatively affect the proliferation and differentiation of OPCs involved in the production of myelin basic protein, which affects the regeneration and repair of the myelin sheath [11,12,13]. Additionally, when these diseases arise, some important inflammatory cells are overactivated and pile up at lesion sites, resulting in the release of many inflammatory factors that include IL-1β, tumor necrosis factor-α, and IL-11. These inflammatory factors can influence the formation of the myelin sheath by decreasing the functional activities of oligodendrocytes in the central nervous system [14,15,16]. The molecular mechanisms behind these pathological effects of IL-1β on oligodendroglia precursor cells remain unclear, but IL-1β is known to promote oligodendrocyte apoptosis [15] and has been reported to obstruct oligodendroglia cells under chronic conditions like cerebral hypoperfusion [17]. Increased levels of IL-1β and its downstream signaling mechanisms have been reported in Alzheimer’s disease and are known to increase subsequent signaling proteins like Toll like receptors (TLR-2), Myeloid differentiation primary response 88 (MyD88), NFκB (nuclear factor kappa-light-chain-enhancer of activated B cells), Cyclooxygenase-1 (COX-1), and Cyclooxygenase-2 (COX-2). The current study aimed at understanding the involvement of IL-1β and its downstream signaling proteins in the MSMS condition. We used HOG and Neuronal Schwan cells, which are principally PNS glia cells, to study the effect of IL-1β in elevating neuroinflammatory markers that may affect pathophysiology and synaptic integrity related to neurotransmission. Though Schwann cells do not play a primary causative or pathogenic role in MS (MS), but they can migrate into the central nervous system (CNS) during the disease process to help repair damaged myelin. As the current study seeks to evaluate the pathological role of IL-1β in MS pathology and the process of demyelination, we used Schwann cells to show the effect on demyelination, along with synaptophysin. We also explored the anti-inflammatory potential of low-cost over-the-counter anti-inflammatory drugs like Ibuprofen and Flurbiprofen in vitro.

## 2. Materials and Methods

### 2.1. Tissue Culture and Maintenance

HOG cells and Rat Neuronal Schwann cells were purchased from ATCC and cultured in Dulbecco’s modified Eagle’s medium (DMEM) (Cat No: 119950065, Thermo Fisher Scientific, Waltham, MA, USA) supplemented to 10% with FBS (fetal bovine serum) (Cat No: 16000044 procured from Thermo Fisher Scientific, Waltham, MA, USA). Cells were treated with/without IL-1β (30 ng/mL) for 48 h and cell pellets were collected by trypsinization. Similarly, cells were pretreated with 100 and 200 ng/mL of IL-1RA prior to IL-1β treatment. Ibuprofen and Flurbiprofen were applied at a concentration of 10 µM 3 h post-treatment along with IL-1β to study the anti-inflammatory effects in vitro.

### 2.2. Western Blot

Total proteins were extracted from HOG cells and Rat Schwann RN100B cells treated with or without IL-1β or IL-1RA along with the drugs 48 h post-treatment. The proteins from these cell lysates were estimated using Bradford reagent (Bio-Rad; Hercules, CA, USA), and 30 μg protein aliquots were electrophoresed for 2 h at 100 V on 4BoltTM 4–12% Bis-Tris Plus (INVITROGEN Thermo Scientific) and transferred to TRANS BLOT. Blots were blocked with BSA blocker for 1 h (Thermo, Waltham, MA, USA) at room temperature and incubated overnight at 4 °C with the primary antibodies depicted in Table 1. After four washes of 5 min each, these membranes were incubated for 2 h at RT with a 1:3000 dilution of HRP-conjugated secondary antibody–goat anti-rabbit IgG or goat anti-mouse IgG with respect to the primary (Cell Signaling Technologies, Danvers, MA, USA), and the blots were developed using an ECL chemiluminescence detection kit procured from Pierce, Waltham, MA, USA. Data were digitized and analyzed with the help of ImageJ software, version 2.0 (NIH). Western blot images were quantified using ImageJ software after subtracting the background with the blot, and all the bands were normalized with GAPDH/Actin. Western blots are representative images from three independent experiments, and individual lanes represent individual biological replicates.

### 2.3. Statistical Analysis

For western blot experiments, the significance of inter-group differences was determined by one-way ANOVA and Bonferroni post hoc analysis using GraphPad Prism 11.1.0. Values were considered significantly different when the two-tailed *p* value was ≤0.05. All the experiments were performed in triplicates with *N* = 3 (biological repeats). The results are expressed as mean ± SD. Significance between untreated and IL-1β- and Ibuprofen-treated cells was determined by one-way ANOVA and the Bonferroni post hoc test, and error bars represent SD. * indicates *p* < 0.05, ** indicates *p* < 0.005, *** indicates *p* < 0.0005, and **** indicates *p* < 0.00005, via one-way ANOVA for an N of 3 biological repeats, represented as individual data points.

## 3. Results

### 3.1. IL-1β Treatment Induces Neuroinflammatory Pathways in HOG and Schwann Cells

**Figure 1:**
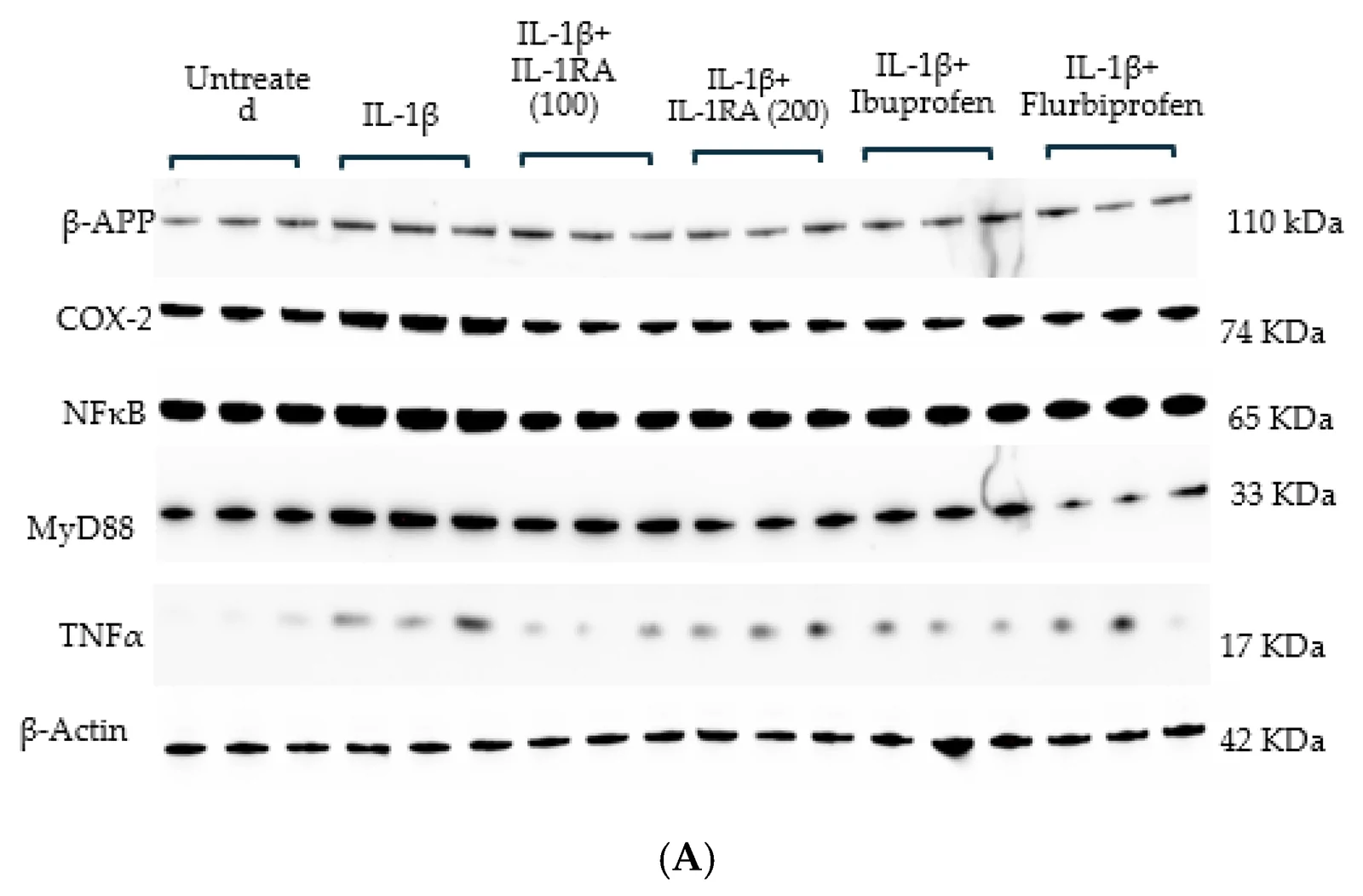

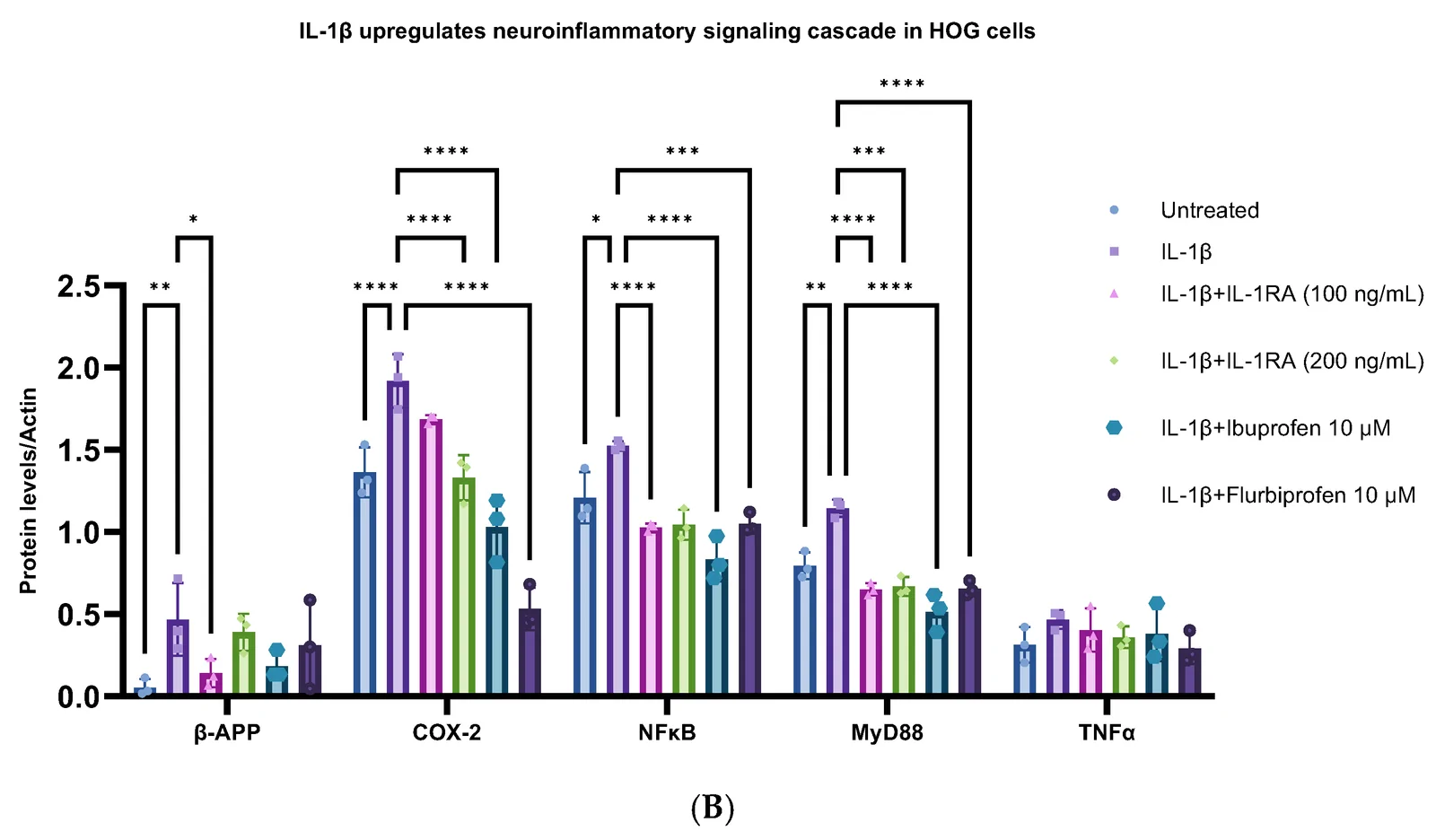
IL-1β upregulates neuroinflammatory signaling cascade in HOG cells. (**A**) Protein levels for neuroinflammatory markers were increased following treatment with IL-1β compared with untreated cells. IL-1RA, Ibuprofen, and Flurbiprofen treatment reduced the protein levels of neuroinflammation (*n*  =  3). (**B**) The bar graph represents the decrease in neuroinflammatory proteins upon Ibuprofen and IL-1RA treatment compared to IL-1β-treated cells. The results are expressed as mean ± SD. Significance between untreated and IL-1β-, IL-1β and Ibuprofen/Flurbiprofen-treated cells was determined by one-way ANOVA with Bonferroni post hoc testing, error bars represent SD. * indicates *p* < 0.05, ** indicates *p* < 0.005, *** indicates *p* < 0.0005, **** indicates *p* < 0.00005, via one-way ANOVA for an N of 3 biological repeats, represented as individual data points.

Here we detail the possible role of the neuroinflammatory cytokine IL-1β in activating a downstream signaling cascade in the two cell types that play leading roles in the pathogenesis of MS, viz., Oligodendrocytes in the brain and Schwann cells in the periphery. The activation of the IL-1β-induced TLR-MyD88-NFκB signaling cascade leads to increases in COX-1 and COX-2 and further increases in IL-1β, as noted in our self-perpetuating “Cytokine Cycle” [18], which is a characteristic feature of other neurodegenerative diseases like Alzheimer’s and Parkinson’s disease [18,19]. IL-1β’s impact in MS is great as it not only downregulates the production of MBP, the principal component of the myelin sheath that allows for the fastest nerve conduction, but it is also the party responsible for the activation of the microglia and macrophages that strip the myelin basic protein sheath from the axon. Further, the IL-1β treatment of these cell lines resulted in elevated levels of neuroinflammatory proteins like MyD88, TNFα, NFκB, COX-1 and COX-2 (Figure 1A,B). Conversely, pretreatment with IL-1RA prior to IL-1β treatment obviated each of the IL-1β-driven increases in signaling genes and proteins, making clear the important role of IL-1β in elevating the pathological changes that manifest as MS. Interestingly, the abatement of the IL-1β effects that was noted with the treatment with cells was also noted following treatment with either Ibuprofen or Flurbiprofen.

### 3.2. IL-1β Induces Inflammatory Pathway Leading to Neurodegeneration in HOG Cells

**Figure 2:**
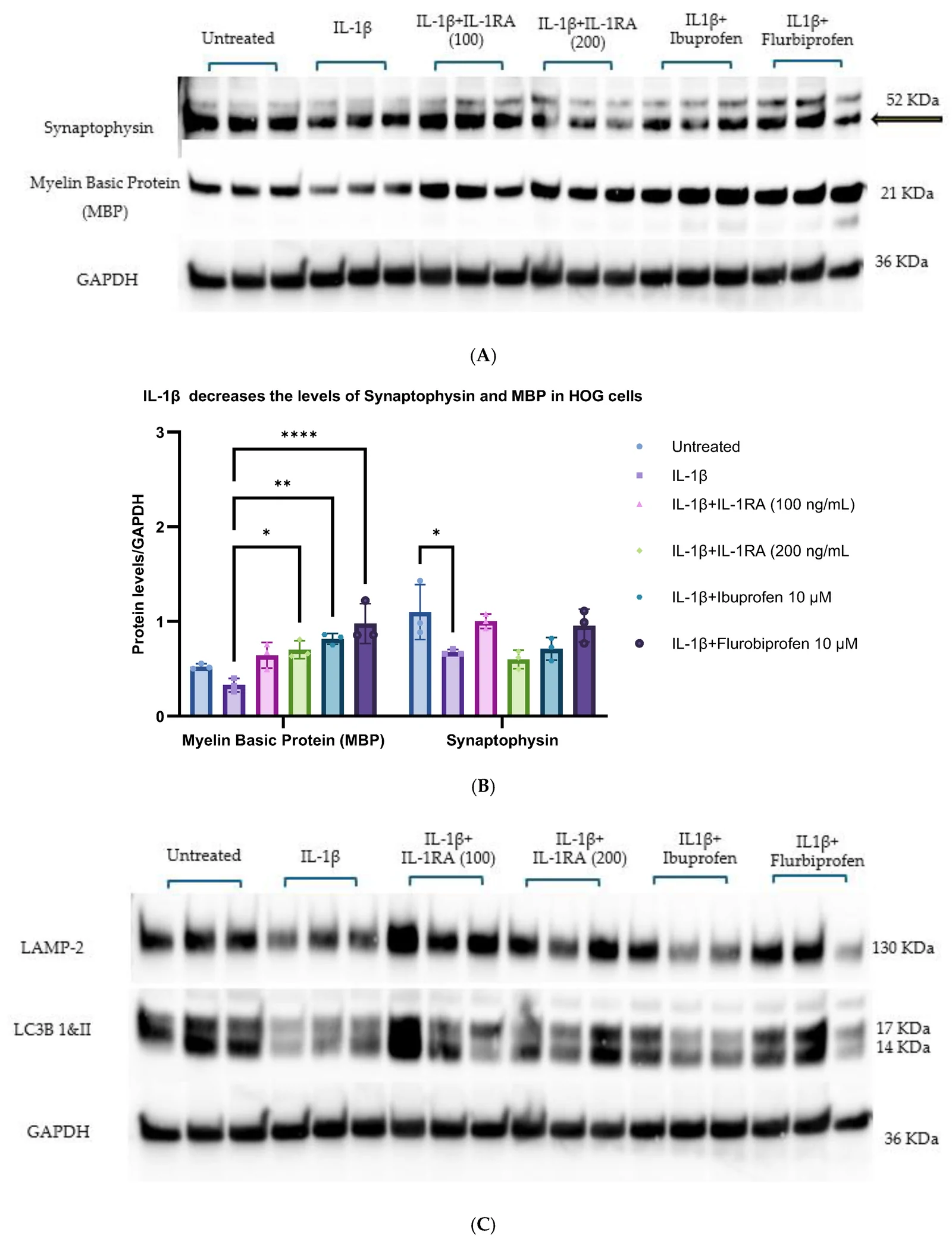

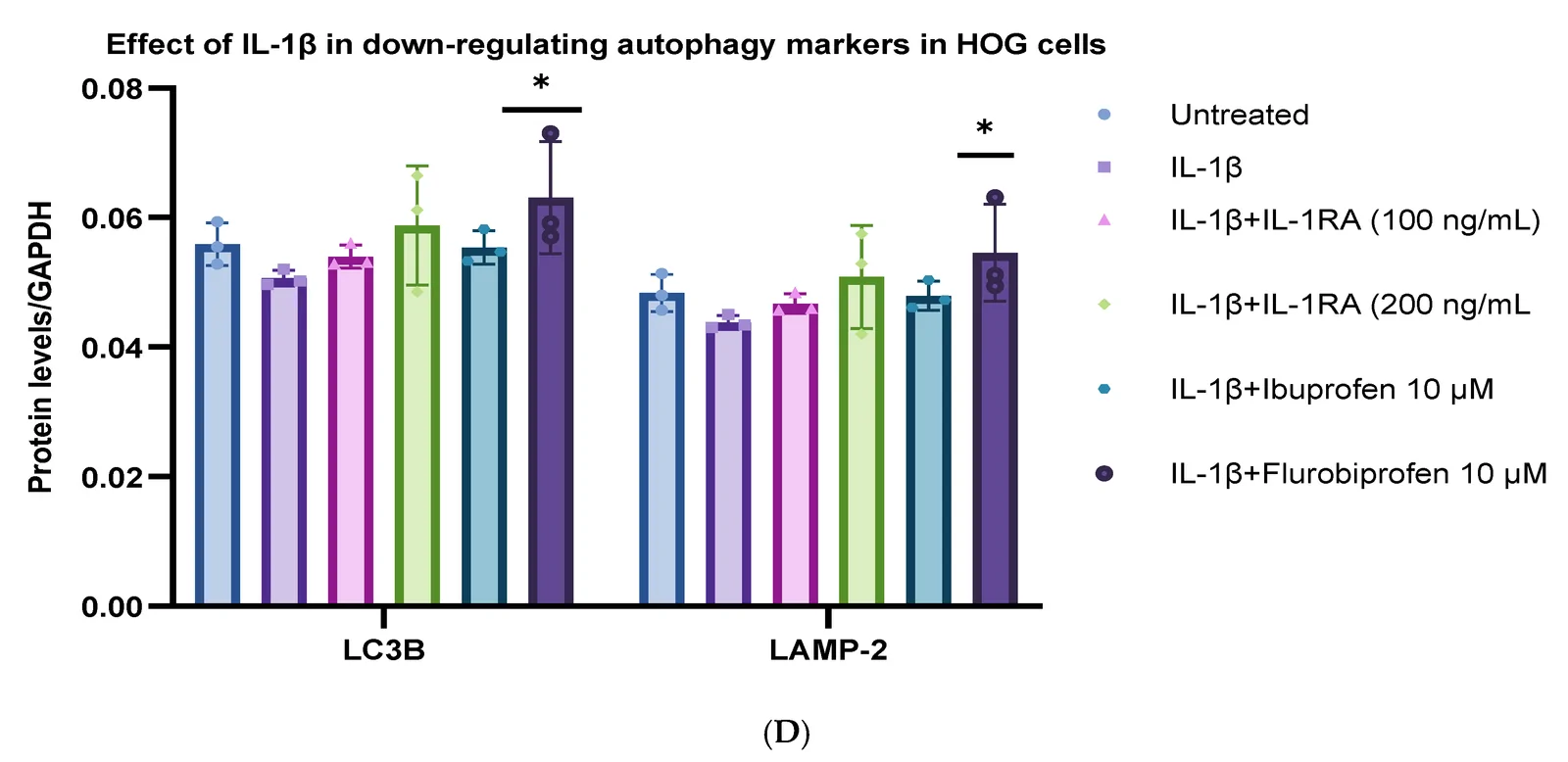
Role of IL-1β in neurodegeneration enacted by decreasing the levels of Synaptophysin and MBP in HOG cells. (**A**) Protein levels for MBP and synaptophysin were decreased under treatment with IL-1β (*n*  =  3) compared to with untreated cells (*n * =  3). (**B**) The histogram represents the results of three independent biological replicates, and the values represented in the histograms are mean ± SD. (**C**) Effect of IL-1β in downregulating autophagy markers in HOG cells. (**D**) The histogram represents the effect of IL-1β in reducing autophagy markers. Results are expressed as mean ± SD. Significance between untreated and IL-1β-, IL-1β and Ibuprofen-treated cells was determined by one-way ANOVA with Bonferroni post hoc test, and error bars represent SD. * indicates *p* < 0.05, ** indicates *p* < 0.005, **** indicates *p* < 0.00005, via one-way ANOVA for an N of 3 biological repeats, represented as individual data points.

Losses of MBP and synaptophysin are the characteristic features of MS [8,9]. In previous reports we showed diminished synaptophysin levels [20] as evidence of the detrimental effects of IL-1β. Here we report the detrimental effect of IL-1β in reducing or diminishing MBP and synaptophysin in HOG cells. After 48 h, the treatment of these cells with IL-1β resulted in a reduction in protein levels (Figure 2A,B) of MBP and synaptophysin in HOG cells. Pretreatment with IL-1 RA prior to IL-1β treatment did not lead to diminished levels of MBP, which clearly indicates the role of IL-1β in this pathological mechanism in MS. Ibuprofen and Flurbiprofen treatment restored and increased the expression of MBP significantly compared to IL-1β-treated cells and synaptophysin in HOG cells, showing that the potential anti-inflammatory effects can reduce the neuroinflammation and neurodegeneration involved in MS pathogenesis.

Diminished autophagy and aggregate accumulation impede the neurotransmission and neuronal stability in both the central and the peripheral nervous system, leading to progressive neurodegenerative diseases like Alzheimer’s and Parkinson’s disease [21,22]. Our previous reports show that the inheritance of the APOEε4 gene is associated with the diminution of autophagy via blocking the production of autophagy-related mRNAs in the brain [23]. Here we report the possible involvement of IL-1β in the autophagic machinery in HOG cells (Figure 2C,D). IL-1β treatment in HOG cells diminished the protein levels of LC3BI&II and LAMP2, which play an important role in the autophagic machinery. These diminished LC3B and LAMP2 levels may impose further cellular stress, leading to increased inflammatory responses due to the buildup of metabolic waste in the cells. Pretreatment with IL-1 RA prior to IL-1β treatment or treatment with Ibuprofen and Flurbiprofen (significantly) restored and increased the expression of these autophagy markers in HOG cells (Figure 2C,D), showing the possible anti-inflammatory effects enacted via reducing neuroinflammation and neurodegeneration in MS pathogenesis.

### 3.3. IL-1β Treatment Induces Neuroinflammatory Pathways That Lead to Neurodegeneration in Schwann Cells

**Figure 3:**
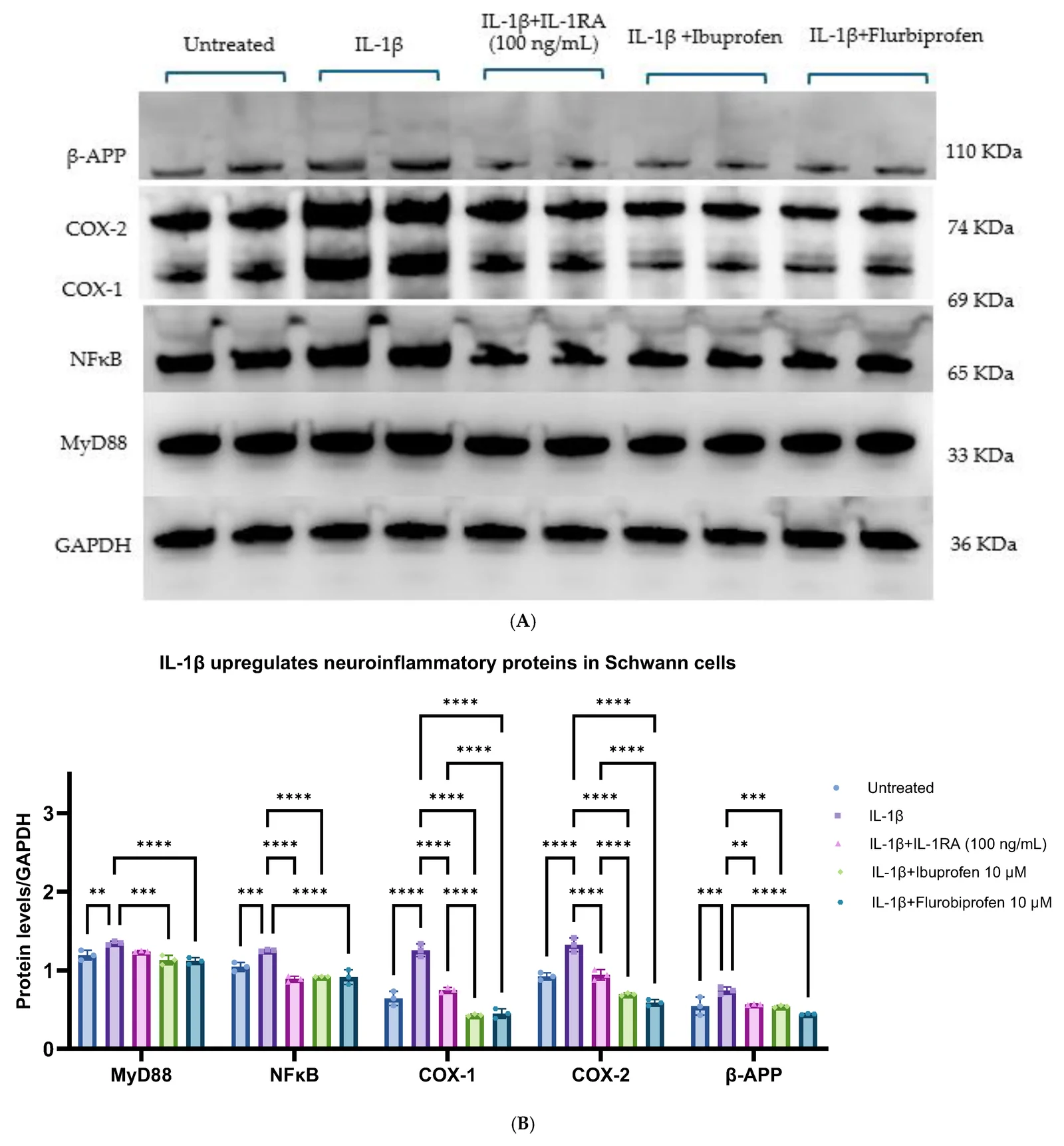

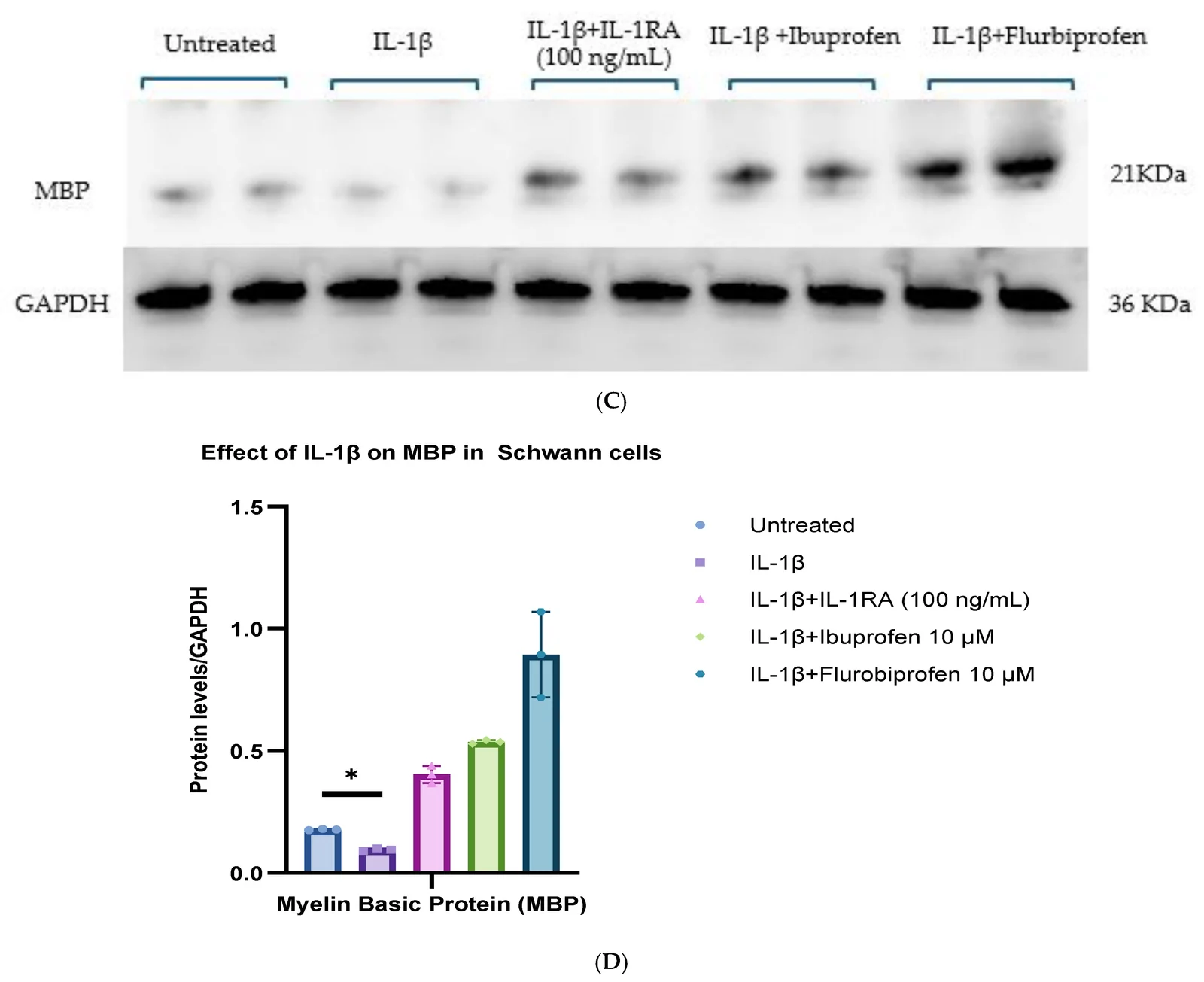
IL-1β upregulates neuroinflammatory proteins in Schwann cells, leading to neurodegeneration. (**A**) Western blot analysis depicting the expression levels of neuroinflammatory proteins in untreated or IL-1β with or without drugs in Schwann cells. (**B**) Histogram representing the expression levels of neuroinflammatory proteins in untreated or IL-1β with or without drugs. (**C**) Western blot analysis showing the expression levels of MBP in Schwann cells in untreated or IL-1β with or without drugs. (**D**) Histogram representing the results of three independent biological replicates. The results are expressed as mean ± SD. Significance between untreated and IL-1β- and Ibuprofen-treated cells was determined by one-way ANOVA with Bonferroni post hoc test, error bars represent SD. * indicates *p* < 0.05, ** indicates *p* < 0.005, *** indicates *p* < 0.0005, **** indicates *p* < 0.00005, via one-way ANOVA for an N of 3 biological repeats, represented as individual data points.

The current study was aimed at understanding the involvement of IL-1β and its downstream signaling proteins in the MS condition. At 48 h post-treatment of Schwan cells using IL-1β, we saw the upregulation of neuroinflammatory proteins like MyD88- NFκB- COX-1 and COX-2 and β-APP (Figure 3A,B) in Schwann cells. Pretreatment with IL-1 RA (100 ng/mL) prior to IL-1β treatment did not manifest the elevated levels of these signaling proteins, which explains the possible role of IL-1β in the pathological mechanism of the MS disease. Ibuprofen and Flurbiprofen treatment reduced these effects, showing their potential anti-inflammatory effect and that they can reduce the neuroinflammation and its detrimental effects in MS pathogenesis.

After 48 h of treatment of Schwann cells with IL-1β, we saw a reduction in MBP levels in the Schwann cells (Figure 3C, p* ≤ 0.05). Pretreatment with IL-1 RA (100 ng/mL) prior to IL-1β treatment did not manifest diminished levels of MBP, which may indicate the potential role of IL-1β in this pathological mechanism in the MS disease. Ibuprofen and Flurbiprofen treatment restored and increased the expressions of MBP and synaptophysin in these cells, showing that their possible anti-inflammatory effects can reduce neuroinflammation and neurodegeneration in MS pathogenesis.

### 3.4. IL-1β Treatment Elevated the JAK-STAT-IL-6 Proteins in HOG and Schwann Cells

**Figure 4:**
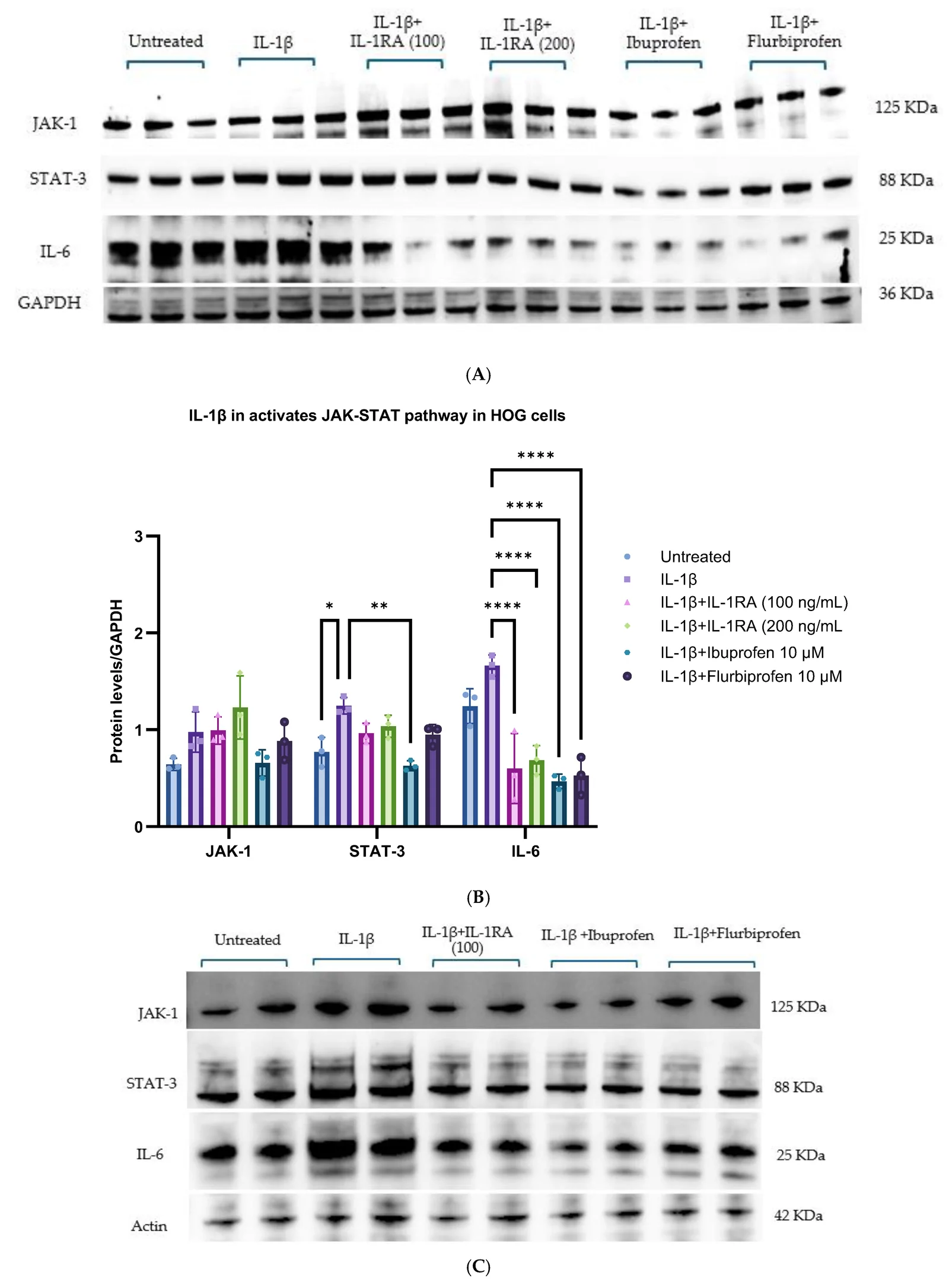

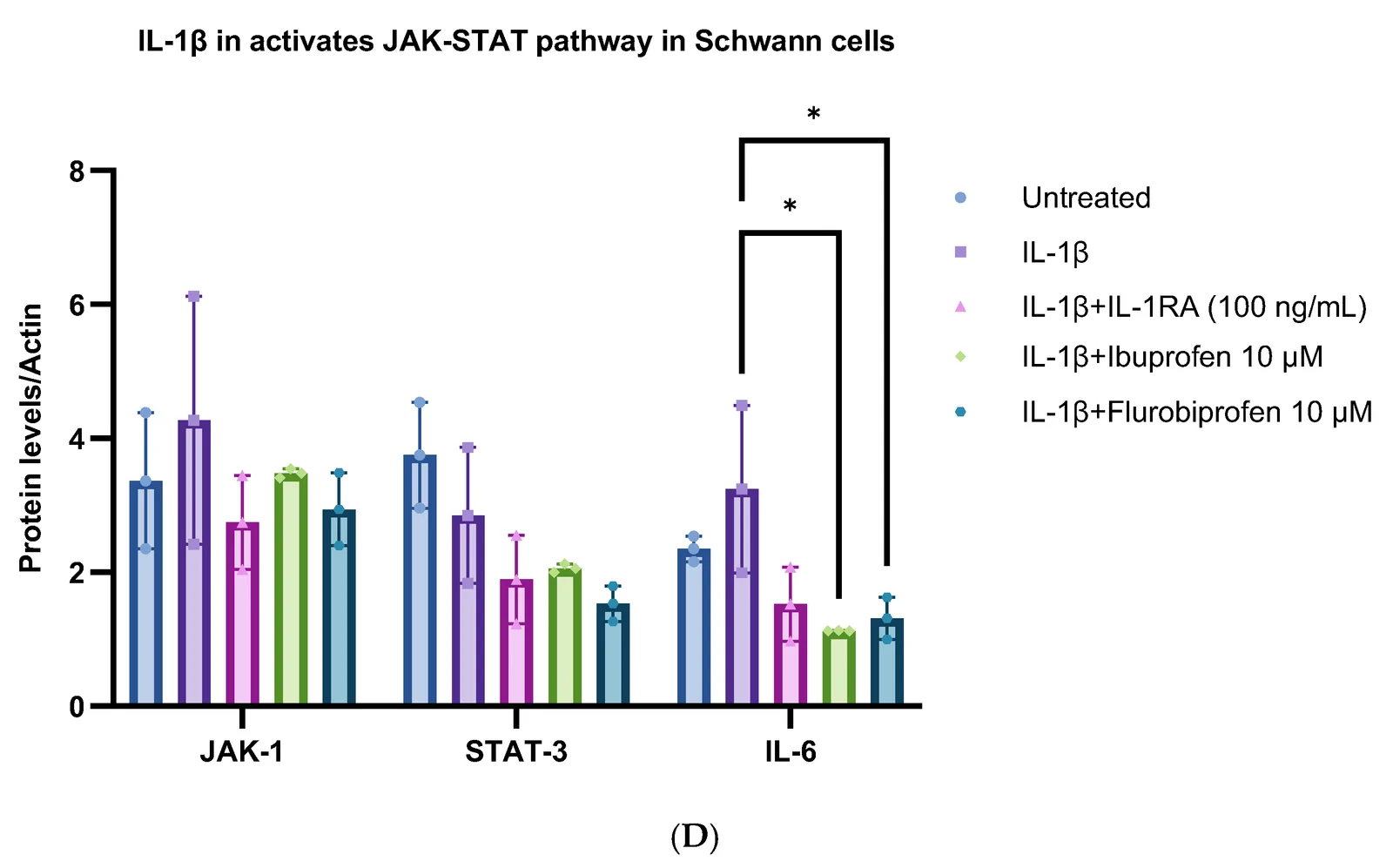
Role of IL-1β in activating JAK-STAT pathway in HOG cells and Schwann cells. (**A**) Protein levels of JAK-1, STAT-3 and IL-6 were increased under the treatment with IL-1β (*n * =  3; biological repeats) compared with untreated cells (*n * =  3). (**B**) Histogram representing the results of three independent biological replicates; values are represented as mean ± SD. (**C**) Role of IL-1β in activating the JAK-STAT pathway in Schwann cells. (**D**) Histogram representing the results of three independent biological replicates. Results are expressed as mean ± SD. Significance between untreated and IL-1β- and Ibuprofen-treated cells was determined by one-way ANOVA with Bonferroni post hoc tests, and error bars represent SD. * indicates *p* < 0.05, ** indicates *p* < 0.005, **** indicates *p* < 0.00005, via one-way ANOVA for an N of 3 biological repeats, represented as individual data points.

The pathological role of the Janus Kinase/Signal Transducer and Activator of Transcription (JAK/STAT) signaling pathway is an important mechanism in the production of proinflammatory cytokine markers like TNF-α, while IL-6 and IL-12, further contribute to the self-perpetuating Cytokine Cycle [18,24] (Figure S1). A 48-h treatment of HOG and Schwann cells with IL-1β resulted in increased levels of JAK-STAT-IL-6 proteins, which are involved in the JAK/STAT signaling pathway (Figure 4A–D, *p* ≤ 0.05). These increases were diminished by pretreatment with IL-1 RA, indicating the possible role of IL-1β in the progression of MS. In this regard, it is interesting to note that in our in vitro experiments, the treatment of HOG and Schwann cells with Ibuprofen or Flurbiprofen reduced the expression levels of the JAK-STAT-IL-6 proteins in vitro, suggesting that therapeutic treatments with Ibuprofen or Flurbiprofen may be helpful in reducing the neuroinflammation and neurodegeneration involved in the pathogenesis of MS.

## 4. Discussion

The work we report here demonstrates the possible ways in which IL-1β plays a role in the pathogenesis of MS by increasing neuroinflammation and the process of the demyelination of axons in the central and peripheral nervous system required for synaptic integrity and neurotransmission. According to recent reports on epidemiological data, the incidence of MS has been progressively increasing over years, affecting approximately 2.9 million individuals worldwide (1 in 3000 people) [25]. The reason for this sustained increase is unknown, but the fact as we show here of the possible role of inflammation, especially the neuroinflammatory cytokine IL-1β, is at least possibly related to the known connection between inflammation and disease pathogenesis in MS.

Although we know that MS is heritable, little else is known about either its origin or why the numbers of MS cases are increasing, or why these increases occur preferentially in particular regions of the world, but as per the results of our in vitro experimental findings using human cells, we do know that the neuroinflammatory immune cytokine Interleukin-1β (IL-1β) may activate the neuroinflammatory pathway leading to the series of steps that could cause the stripping of the myelin sheath from axons of neurons in the oligodendrocytes, and of neurons in the periphery with information destined for synapses in the body. For instance, as a beginning step, IL-1β activates the myelin-reactive T-helper cells TH1 and TH17, which, in turn, are responsible for the initial stripping of the insulating sheath that normally serves to insulate long axons and ensure the fast and secure conduction of neuronal information [26]. Over time, this initial IL-1β-driven stripping leads to the degeneration of the nerve, the loss of specific and important information, and ultimately the physical disability related to MS [27]. Over and above the loss of timely nerve signal information, this possible IL-1β-driven loss of not only the axonal sheath but also the sheath’s Nodes of Ranvier, which hinders the fastest conduction of information along the axon IL-1β, also lowers the synthesis of the myelin basic protein, the primary protein of the myelin sheath [28]. These events dramatically slow functional signals from the brain to the periphery, allowing for the interruption of information and the introduction of irrelevant or mixed signals that may be related to patient complaints of false sensations, such as heat or cold [29].

In a randomized open-label study comparing naproxen, acetaminophen and Ibuprofen, the potential use of non-steroidal anti-inflammatory drugs for controlling side effects related to the initiation and ongoing use of interferon beta-1a for relapsing–remitting MS been shown to be effective [30]. In our pioneering work in 1989 [31] where we showed for the first time evidence of the leading role of neuroinflammation, with IL-1β as a diagnostic marker protein in neuropathogenesis, in Alzheimer’s and Downs Syndrome, we provided a template for the study of other diseases that are characterized by neuronal stress and glial activation. Recently we reported that elevated levels of IL-1β and its downstream signaling cascade TLR-MyD88-NFκB lead to increases in COX-1 and COX 2 in both conditions. This is an important downstream consequence of one of the cardinal signs of Alzheimer Aβ plaques, related to IL-1β-induced synthesis and the activation state of MAPKp38, an important kinase for the phosphorylation of both tau (related to the neurofibrillary tangles that are a cardinal feature of Alzheimers) and of a-synuclein (related to the Lewy bodies diagnostic of Parkinson’s disease (PD), multiple-system atrophy (MSA), and Lewy Body dementia) [32]. IL-1β and TNFα treatment increased the levels of αsyn protein in primary neurons in a dose-dependent manner, which resulted in the activation of the TLR-MyD88-NFκB pathway [33]. Here we add further evidence of a possible pathological role of IL-1β in driving the neuropathogenesis of MS via IL-1β-driven inflammatory signaling cascades that may show deleterious effects in the production of myelin basic protein, a mainstay in the formation and maintenance of the myelin sheath that wraps the long axons from cortical neurons to synapses throughout the body.

Here, we sought to understand the principal actuate of the report in patients of the loss of the myelin sheath. Upon discovering that neuroinflammation, particularly related to cytokine IL-1β and its downstream pathways, was a critical actor, we sought to determine if over-the-counter remedies like Ibuprofen, which is known to decrease the risk of developing Alzheimer’s and Parkinson’s disease [34] via its quelling of IL-1β-related neuroinflammation, might be effective in reducing the effect in an in vitro setting, and whether such intervening actors might result in restored conduction, and over time counteract or slow the chronic disability of the patient. Animal models of MS (MS) have demonstrated that nonsteroidal anti-inflammatory drugs (NSAIDs) like Ibuprofen can exert beneficial anti-inflammatory and neuroprotective effects by inhibiting cyclooxygenase (COX), thus reducing oligodendrocytes apoptosis, demyelination, and motor dysfunction pathways [35]. Our results support the above evidence that the in vitro treatment of HOG and Schwann cells with IL-1β resulted in increased levels of COX-1 and COX-2, which were quelled after treating Ibuprofen and Flurbiprofen. Genome-wide association studies have reported altered NFκB responses due to the enhanced expression of NFκB itself inducing a 20-fold increase in NFκB p50 and reductions in several negative regulators of NFκB [36]. Here we report that the increased expression of NFκB upon IL-1β treatment may suggest the possible involvement of this cytokine in the mediation of inflammatory responses in MS. However, the functional effects and modes of activation of the NF- and NFκB proteins in respect to their phosphorylated/dephosphorylated states are yet to be elicited. More detailed studies using animal and other higher-level disease models may be undertaken to understand these effects in greater detail.

Although some potential, strong and effective drug treatment strategies are available, they are costly and not readily available. Our in vitro study’s results, derived using low-cost over-the-counter anti-inflammatory drugs, are consistent with the idea that their use may act to reduce neuroinflammation and its signaling protein lines COX-1 and COX-2, which may help the axon sheath by protecting synaptophysin and MBP. However the functional effects and modes of activation of the STAT protein in respect to their phosphorylated/dephosphorylated states are yet to be outlined. More detailed studies using animal and other higher-level disease models may be undertaken to help us understand these effects in greater detail.

The limitations of the current study include the use of immortalized/tumor-derived HOG cells to study the complex mechanisms of MS. Currently; our results are completely based on our in vitro findings derived using HOG and Schwann cells. The lack of an animal/EAE model limits our conclusions, and a detailed study using an animal/EAE model may help to substantiate our findings. Further, the use of functional myelination/remyelination assays using primary human oligodendrocytes/neurons of CNS origin may support our interpretations.

## 5. Conclusions

IL-1β could have a detrimental effect, facilitating the neuropathogenesis seen in Alzheimer’s and Parkinson’s diseases by activating neuroinflammatory proteins like MyD88, NFκB, COX-1 and COX-2 in vitro. IL-1β may be involved in the diminished levels of MBP and synaptophysin, thus affecting synaptic integrity and signal transduction. The anti-inflammatory drugs Ibuprofen and Flurbiprofen potentially play roles in modulating neuroinflammation, and may improve synaptic integrity and signal transduction in MS. Further detailed studies using animal/EAE models may be undertaken to help us understand the potential therapeutic applications of these molecules in MS.

## Figures and Tables

**Table 1:** Primary antibodies used in the study.

Antibody	Species	Cat. No.	Company
TNF-alpha	Rabbit mAb	bsm-55603r	BIOSS, Woburn, MA, USA
MyD88	Rabbit mAb	ab133739	Abcam, Cambridge, MA, USA
NF-κB p65	Rabbit mAb	ab32536	Abcam, Cambridge, MA, USA
COX-1	Rabbit mAb	ab109025	Abcam, Cambridge, MA, USA
COX-2	Rabbit mAb	ab179800	Abcam, Cambridge, MA, USA
βAPP	Rabbit mAb	ab32136	Abcam, Cambridge, MA, USA
Myelin Basic Protein	Rabbit mAb	ab7349	Abcam, Cambridge, MA, USA
Synaptophysin	Rabbit mAb	#36406	Cell Signaling Technology, Danvers, MA, USA
JAK-1	Rabbit mAb	#3344	Cell Signaling Technology, Danvers, MA, USA
STAT-3	Mouse mAb	#9139	Cell Signaling Technology, Danvers, MA, USA
IL-6	Rabbit mAb	#12912	Cell Signaling Technology, Danvers, MA, USA
LC3B	Rabbit mAb	NB6001384	Novus Biologicals, Centennial, CO, USA
LAMP-2	Rabbit mAb	#34141	Cell Signaling Technology, Danvers, MA, USA
β-Actin	Rabbit mAb	#4970	Cell Signaling Technology, Danvers, MA, USA
GAPDH	Mouse mAb	sc47724	Santa Cruz Biotechnology, Inc., Dallas, TX, USA

## Data Availability

The original contributions presented in this study are included in the article and Supplementary Material. Further inquiries can be directed to the corresponding author.

## Supplementary Materials

The following supporting information can be downloaded at: https://www.mdpi.com/article/10.3390/cimb48090953/s1.
