# Supplementary material for: Interleukin-1β-Induced Inflammatory Signaling and Myelin-Related Alterations in Oligodendroglia and Schwann Cells in Multiple Sclerosis: Modulatory Effects of Ibuprofen and Flurbiprofen
Source: Curr Issues Mol Biol. 2026 Sep 18;48(9):953. doi: 10.3390/cimb48090953 (PMC13605145; doi:10.3390/cimb48090953)
Supplement: Supplementary file 1 [file cimb-48-00953-s001.zip › cimb-4533930-supplementary.pdf]

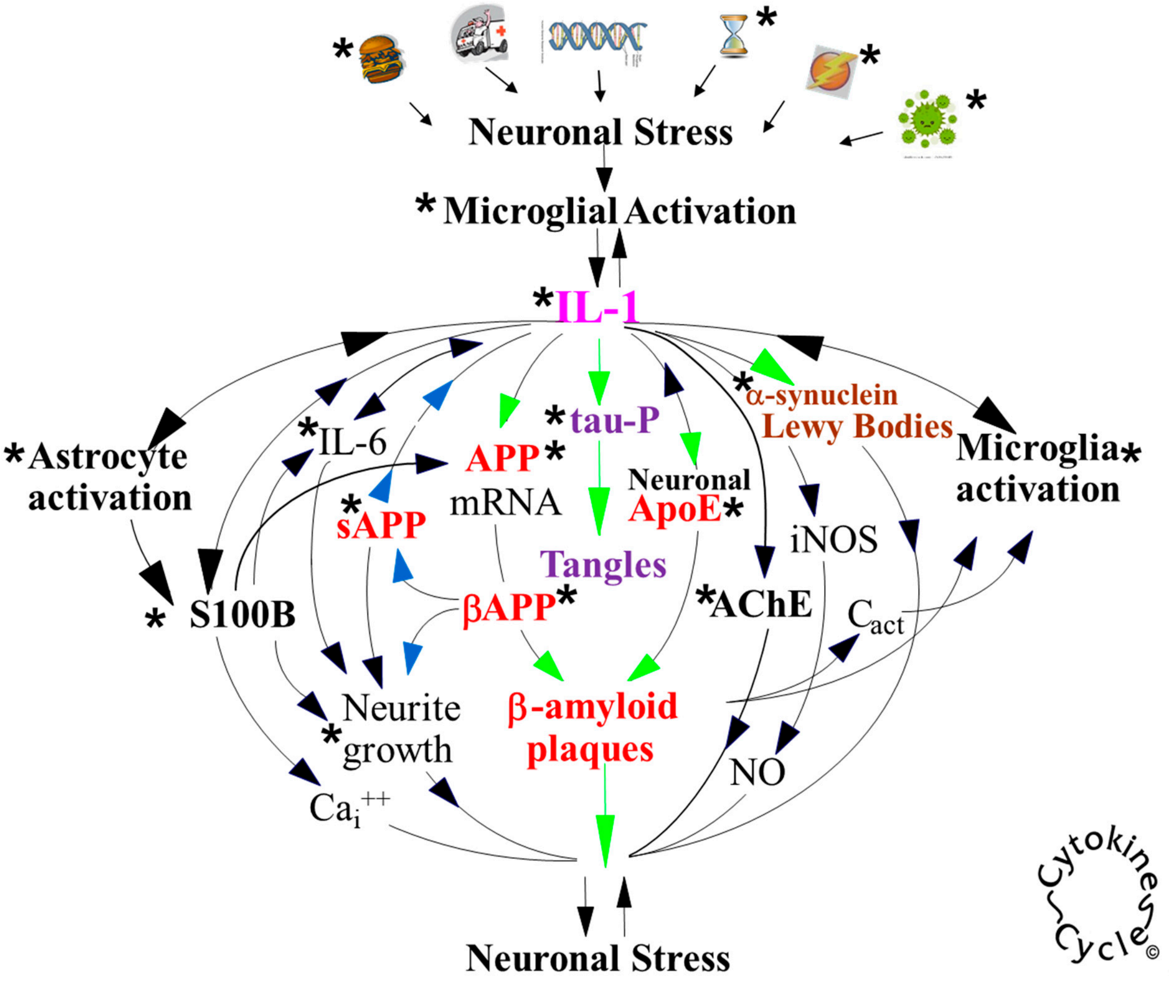

Figure S1. Self-perpetuating cytokine cycle. IL-1β acting as potential driver of components in neuropathogenesis.
